# De-identification of medical imaging data: a comprehensive tool for ensuring patient privacy

**DOI:** 10.1007/s00330-025-11695-x

**Published:** 2025-06-07

**Authors:** Moritz Rempe, Lukas Heine, Constantin Seibold, Fabian Hörst, Jens Kleesiek

**Affiliations:** 1 Institute for AI in Medicine (IKIM), University Hospital Essen, Essen, Germany; 2 Cancer Research Center Cologne Essen (CCCE), University Medicine Essen, Essen, Germany; 3 Department of Physics of the Technical University Dortmund, Dortmund, Germany; 4 RACOON Study Group, Site Essen, Essen, Germany; 5 German Cancer Consortium (DKTK), Partner Site Essen, Essen, Germany

**Keywords:** Data privacy, Medical machine learning, Medical imaging

## Abstract

**Objectives:**

Medical imaging data employed in research frequently comprises sensitive Protected Health Information (PHI) and Personal Identifiable Information (PII), which is subject to rigorous legal frameworks such as the General Data Protection Regulation (GDPR) or the Health Insurance Portability and Accountability Act (HIPAA). Consequently, these types of data must be de-identified prior to utilization, which presents a significant challenge for many researchers. Given the vast array of medical imaging data, it is necessary to employ a variety of de-identification techniques.

**Materials and methods:**

To facilitate the de-identification process for medical imaging data, we have developed an open-source tool that can be used to de-identify Digital Imaging and Communications in Medicine (DICOM) magnetic resonance images, computer tomography images, whole slide images and magnetic resonance twix raw data. Furthermore, the implementation of a neural network enables the removal of text within the images.

**Results:**

The proposed tool reaches comparable results to current state-of-the-art algorithms at reduced computational time (up to × 265). The tool also manages to fully de-identify image data of various types, such as Neuroimaging Informatics Technology Initiative (NIfTI) or Whole Slide Image (WSI-)DICOMS.

**Conclusion:**

The proposed tool automates an elaborate de-identification pipeline for multiple types of inputs, reducing the need for additional tools used for de-identification of imaging data.

**Key Points:**

***Question*** *How can researchers effectively de-identify sensitive medical imaging data while complying with legal frameworks to protect patient health information?*

***Findings*** *We developed an open-source tool that automates the de-identification of various medical imaging formats, enhancing the efficiency of de-identification processes.*

***Clinical relevance*** *This tool addresses the critical need for robust and user-friendly de-identification solutions in medical imaging, facilitating data exchange in research while safeguarding patient privacy.*

**Graphical Abstract:**

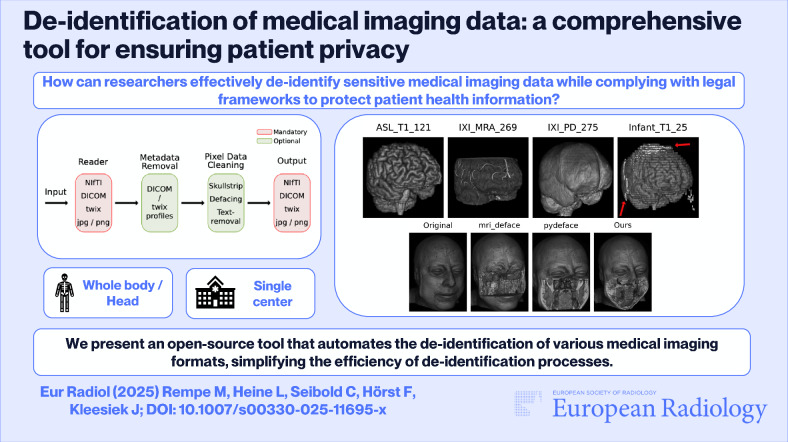

## Introduction

Working with real-world medical data confers a substantial advantage upon research in comparison to artificially generated data, as it can reflect the actual distribution of data within our world. Nevertheless, using patient data raises the issue of privacy. Medical data—including imaging scans—contain sensitive information which has to be strictly protected [1]. Before working with medical imaging data or distributing it to other researchers, this information must be de-identified. One example is the RACOON project, which is a consortium of multiple German research sites that exchange clinical data. This motivates the necessity for a uniform de-identification pipeline to ensure that data contributed by individual sites adheres to predefined conventions and is straightforward to implement into existing workflows.

This task can become tedious with different types of imaging data. Clinicians have several medical imaging techniques at their disposal, including magnetic resonance imaging (MRI) and computer tomography (CT), both of which have different ways of storing the resulting data. Some of these storage formats are Digital Imaging and Communications in Medicine (DICOM) [2], the Neuroimaging Informatics Technology Initiative (NIfTI) [3] format, or raw data formats such as Siemens twix (.dat) [4]. Each of these formats has different attributes and information stored, requiring the use of several different tools to de-identify this range of data, leading to potential confusion and errors.

Although de-identification and anonymization are often used interchangeably, they differ in that de-identification removes or conceals only direct identifiers, whereas anonymization ensures that no data can be traced back to an individual in any manner. Pseudonymization, in comparison, is replacing the personal data with reversible code, which can be recovered with a corresponding key. Thus, the proposed tool is focused on de-identification.

Most de-identification tools focus on the stored metadata. Medical imaging data contains more sensitive data than just the meta information stored. In particular, brain scans can lead to privacy violations, as they can be subjected to full facial reconstructions if they contain the skull [5]. With the advent of machine learning, facial reconstruction and general identification of patients based on medical imaging data achieve more accurate results [6]. Thus, in addition to meta information de-identification, further steps, such as skull-stripping, are needed, which removes the skull from the scan [7]. To address the challenges mentioned above, we present a novel tool that combines multiple options for de-identification. While no de-identification is perfect, this tool covers all necessary steps for scientific de-identification, as it provides outputs that are not identifiable without larger efforts of re-identification. Our approach is the best effort toward anonymization while maintaining practicality.

### Previous work

With the rise of digital medical imaging files, the need for de-identification tools arose. Since then, several tools have been proposed and established for different file formats and applications. Most of these tools perform a specific task, often for only one file format.

Mason et al [8] propose a DICOM metadata de-identification tool using the *pydicom* framework. The *Freesurfer* library [9] contains tools for skull-stripping as well as defacing of MRI NIfTI data. These two tools already show a major problem for practitioners in the medical domain: there is a tool for almost every de-identification task, but not a tool that works for all the major file and imaging formats. Additionally, tools like the deface algorithm by *Freesurfer* can be very time-consuming, slowing down post-processing pipelines.

When it comes to tools for WSI (Whole Slide Image) data, the literature is rather scarce, especially when looking at open-source software. Bisson et al [10] presented in their work an open-source C-based tool for WSI de-identification that works on a variety of native WSI file formats. Nevertheless, it does not work on WSI DICOM files.

## Materials and methods

### Medical imaging data types

There are numerous different ways to store the acquired digital data, due to the wide range of medical imaging techniques, such as magnetic resonance imaging (MRI), computer tomography (CT), ultrasound imaging (US) and WSI. The most popular data types are [4]:

- Analyze
- DICOM
- NIfTI
- MINC [11]
- JP(E)G, PNG

While Analyze, MINC, and NIfTI are data formats used for research purposes, DICOM (and derivatives, such as DICOM-WSI in pathology) is the file format mainly used in clinical practice. Additionally, most post-processing software is compatible with these two formats, which is not the case for Analyze and MINC. JP(E)G and PNG are file formats mainly for (clinical) photographic images, for example, of wounds or other surface inspections. In addition to the number of different file format standards, there are raw data formats from different vendors, an example of which is the twix raw data format for MRI data from Siemens.

### De-identification

Medical image de-identification consists of multiple separate tasks:

- Metadata anonymization
- Defacing
- Skull-stripping
- Text removal
- WSI de-identification

For this work, we differentiate between personal and sensitive patient data. While personal data includes patient-specific information, such as name, home address or identification number, sensitive data is a broader category including data such as ethnicity, sex or age. Depending on the use case, the proposed tool allows the use of different de-identification profiles. Users should be aware of the implications these differences might have on, e.g., bias in studies.

Metadata anonymization aims to remove all person-related meta information in the data header, such as name, sex, date of birth, or diagnosis. Because the different data types have different metadata headers, this task spans a wide range of variation in what data needs to be removed. For DICOM images, the standard defines a guideline on how to handle metadata [12]. In short, a basic profile on how different metadata information should be handled. At the time of writing, the standard defines ten additional profiles that extend the basic profile and allow limited preservation of some metadata tags if needed. We provide these profiles to be used out of the box to ease the burden of manually checking for conformity. In addition, users may add profiles tailored to their specific needs and legislation.

Defacing is the task of removing the face from a medical image scan, while leaving the rest of the image intact. Common tools for this task are *mrideface* (*Freesurfer*) [7] or *pydeface* [13]. While defacing does not remove the whole skull, the aim of skull-stripping is to only output the brain, thus preserving relevant anatomical information. There are procedures that explicitly require that sensitive structures, such as the eyes, be preserved. In such cases, skull-stripping may not be a viable option. This approach is more common and thus more researched, with the most popular tools being *SynthStrip* [14] or the *Brain Extraction Tool* (BET) [15]. Isensee et al utilize deep learning for brain extraction with their *HD-BET* model [16]. While these tools are well-introduced in clinical and research practice, they do come with downsides. One major issue is the long computation times of all approaches. *mrideface* and *pydeface* both need more than a minute per volume, making it unfeasible for real-time usage.

**Fig. 1:**
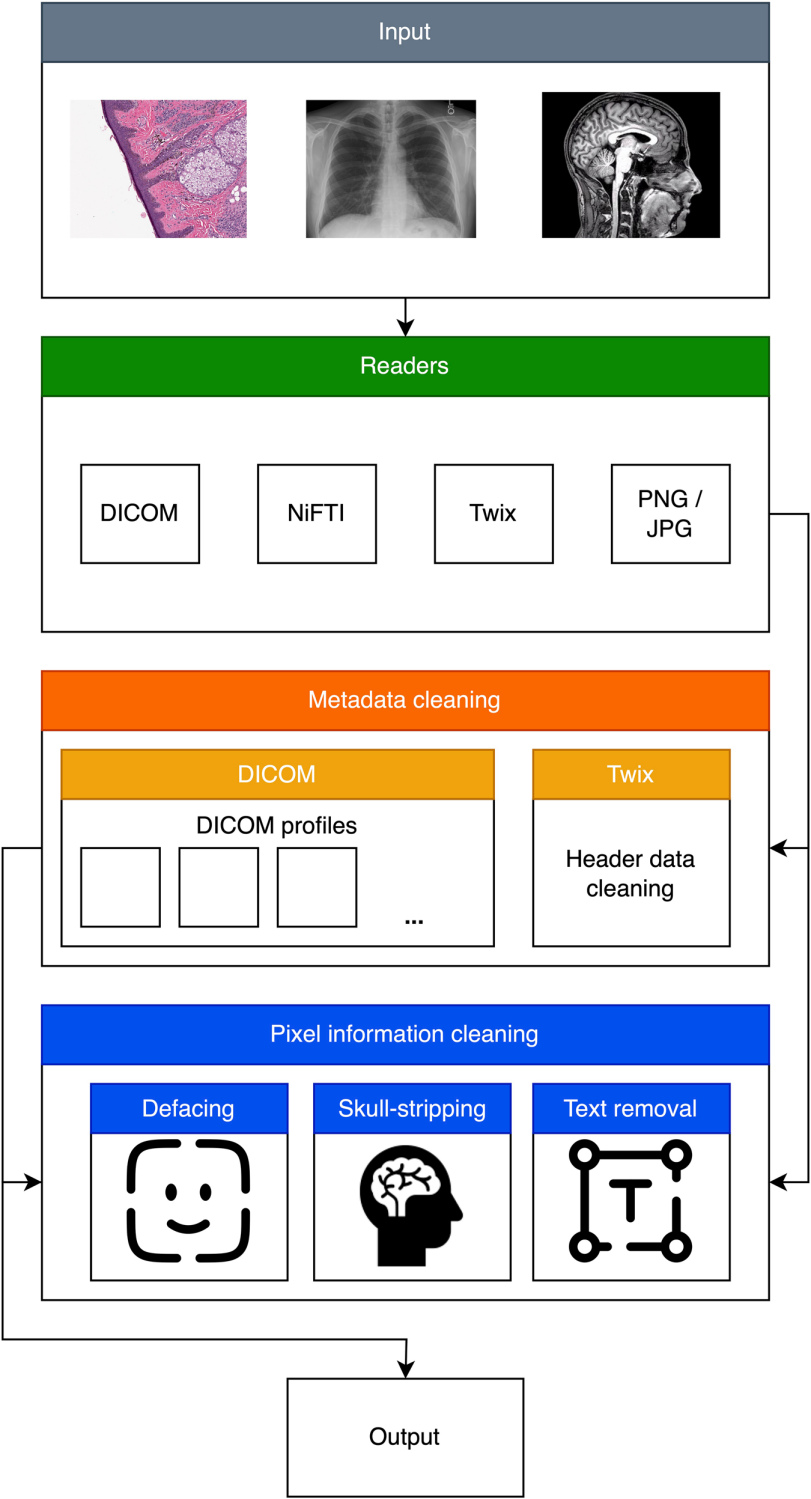
Overview of the proposed de-identification tool. The input data is first read, specific to its data type. Different optional de-identification steps can be performed. Metadata removal or pixel data cleaning, including skull-stripping, defacing or text removal, can be performed for all common medical imaging data types

The proposed tool is able to de-identify a wide range of data types, including MRI, CT and WSI DICOM files, as well as NIfTI and Siemens MRI raw twix data files (.dat). The DICOM files are de-identified by modifying the metadata according to the predefined ruleset and then performing skull-stripping. The NIfTI file header is anonymized by removing the content of the text fields “descrip” and “intent_name,” which are the only patient-related metadata information in a NIfTI header. Because the MRI raw data does not display image domain data, but is in the k-Space, this data does not undergo skull-stripping, but its header is anonymized. An overview of the proposed tool is shown in Fig. 1.

### Technical requirements

We provide the proposed tool as a Python3 Command Line Interface (CLI) application as well as a standalone docker container, which can be found at https://hub.docker.com/r/morrempe/hold. We additionally make our code publicly available at https://github.com/TIO-IKIM/medical_image_deidentification. The technical frameworks used can be found in Table 1.

### Datasets

For the wide variety of different de-identification tasks, multiple different datasets are necessary for training and testing. For training of the skull-stripping approach, we used three publicly available datasets:

- Neurofeedback Skull-stripped (NFBS) repository [17]
- Calgary-Campinas-359 (CC-359) dataset [18]
- Synthstrip validation dataset [14]

The NFBS dataset consists of 125 T1-weighted scans, with a resolution of 1 mm^2^, of subjects between 21 to 45 years old. The ground truth, consisting of skull-strip segmentations, is generated with the STAPLE algorithm [18], which combines ground truth from different annotators. The CC-359 dataset consists of 354 T1-weighted individual subjects from different scanner manufactures (Siemens, GE & Philips). The Synthstrip validation dataset is a publicly available subset of the data used to train the Synthstrip dataset and consists of 24 volumes of different sequences and modalities, including MRI, CT and PET. For testing we use the Synthstrip test dataset. The authors already give a test-split, which includes different modalities, such as diffusion weighted imaging (DWI), T1, T2 and FLAIR, as well as infant subjects. Thus, the total test dataset consists of 558 subjects, with different amounts of slices per volume.

Training of the proposed defacing algorithm was performed on a custom dataset containing 17 volumes of the GBM dataset and 20 volumes of the Synthstrip dataset. We chose the volumes such that a wide variety of modalities is covered in the dataset. Testing is conducted on 18 subjects of the “IXI-T1” cohort in the Synthstrip dataset, as well as 3 infant subjects. The ground truth for the training of the defacing dataset is created by applying the *pydeface* algorithm, as it yielded better results than the *mrideface* algorithm.

The proposed text removal algorithm is tested on an internal ultrasound imaging dataset, consisting of 262 2D scans of different anatomies, including the kidney and liver.

### Experiments

**Fig. 2:**
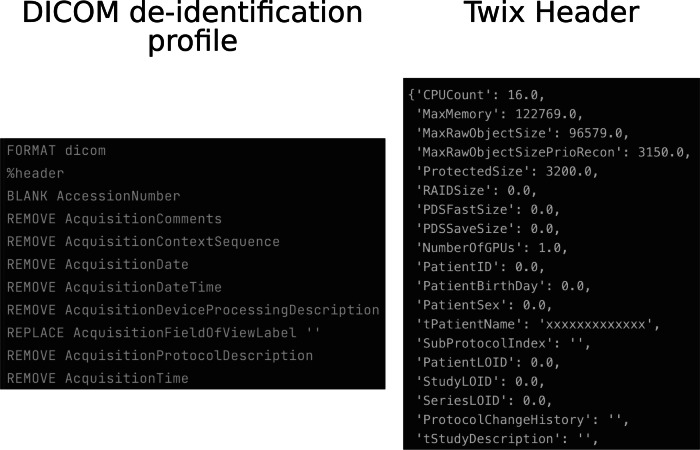
Exemplary excerpt of a DICOM de-identification profile (left) and part of an exemplary anonymized twix header (right). All patient-related information is anonymized by either replacing the values with zeros or ‘x’

Twix data contains multiple headers. While its DICOM header, saved as [‘hdr’], can be anonymized like any other DICOM header, the much larger header [‘hdr string’] is often overlooked, but contains all the same information as the general header, as well as a detailed overview of the scan settings. If de-identification of twix data is only performed on the DICOM header, all the information contained in the latter will be pasted back into [‘hdr’] when saving the “anonymized” file. This brings up the necessity and chances of the proposed tool, which scans the hdr string for all information that needs to be anonymized and replaces it accordingly. Part of an exemplary anonymized twix header can be seen in Fig. 2 (right). The patient related metadata is either replaced with zeros, such as “PatientID” and “PatientBirthday,” while text fields, such as “tPatientName” are replaced by “x.”

For skull-stripping and defacing, a 3D MedNext [19] is used, which is not pretrained. In previous studies, this model showed results on medical imaging data superior to commonly used architectures. Each input NIfTI file is first reoriented into the (‘R’, ‘A’, ‘S’) orientation, first dimension pointing to the right-hand side of the head, second dimension toward the Anterior aspect of the head and the third dimension toward the top of the head. The data is then converted into pytorch tensors. In case of DICOM input data, the data is first converted into NIfTI files via the *pydicom* library. For training, we used augmentations, including randomly inserted noise or spiking, as well as deformations, to provide our models with the ability to also work with data that might contain artifacts. All augmentations are implemented with torchIO [20], a Python library specialized in the preprocessing of medical images. The initial learning rate is set to 5e − 4 with a cosine annealing schedule and a DICE loss function. All input data is normalized and standardized. A batch size of one is used, and the input is resized to a shape of [64, 224, 224]. Training was performed on a single NVIDIA A6000 GPU with 48 GB of graphics memory.

**Fig. 3:**
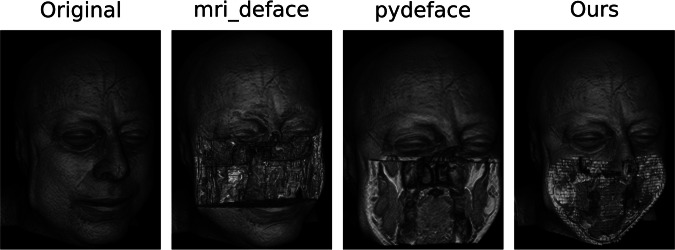
Comparison of the defacing results of different defacing algorithms. While the result of *pydeface* and the proposed algorithm are similar, *pydeface* additionally cuts off the shoulder region of the scan, while taking 260 times longer on average than the proposed algorithm. The shown face is from the publicly available Synthstrip dataset

To compare our defacing algorithm with other existing state-of-the-art algorithms (Table 1), we developed a “defacing-score.” The output data of the deface algorithm is first loaded into the medical image viewer *ITK-SNAP* [21] to generate a volumetric view of the scan. The front-facing view of this volume is then saved as an image. An exemplary output of our defacing model can be seen in Fig. 3. By applying the face recognition model by Geitgey [22], we can then distinguish between correct and flawed defacing results. The defacing score is calculated by the number of defaced scans, which the face recognition model cannot classify as faces anymore. The used function “face_detection” does not have relevant tunable parameters and thus is not prone to biased fitting. For the comparison of the skull-stripping algorithms, we use the commonly used DICE score. As computation time is crucial for real-time algorithms, we additionally track the computation time per volume for each algorithm.

**Table 1:** Overview of third-party modules

Name	Version
pandas	2.0.1
tqdm	4.64.1
torch	2.2.0
timm	0.9.2
numpy	1.23.5
torchvision	0.17.0
PyYAML	6.0
pydicom	2.3.1
deid	0.3.21
torchmetrics	0.11.4
pathlib	1.0.1
nibabel	5.2.1
scipy	1.13.1
torchio	0.19.6
tesseract	5.4.1

**Fig. 4:**
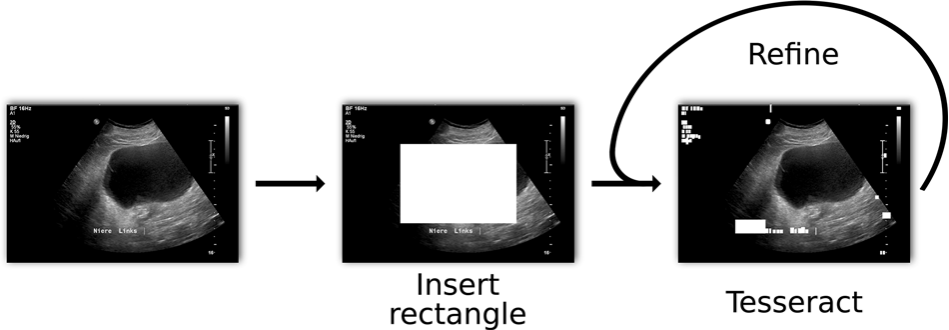
Proposed text removal pipeline at the example of an ultrasound image. By first inserting a rectangle in the center of the scan, Tesseract focuses on the text on the side of the image. Possible texts in the middle of the image are then removed in further iterations

By utilizing the *Tesseract* [23] algorithm for text detection, we propose a novel pipeline for text removal in medical images. To reach consistent de-identification results, the proposed method first inserts a white rectangle in the center of the input image. That way, *Tesseract* focuses on the border of the scans, which holds the majority of the text information of medical scans. In a second iteration, the rectangle is removed, and the detection algorithm is applied another time on the already processed image. For the final output, all detected boundary boxes containing letters are then filled and thus covered. The proposed text removal pipeline can be seen in Fig. 4. The method is tested on 262 2D ultrasound scans.

DICOM-WSI data is saved in a hierarchical structure, consisting of the different zoom-stages of the image, an overview image and a label image. The label image contains information to identify the slide and patient. We remove this information by overwriting the whole pixel array with zeros. Often, the overview image also contains some kind of label that needs to be de-identified, which can be removed by overwriting the left side of the overview image. Additionally, to the image information, each DICOM file contains metadata that has to be removed. In case of converted DICOM files from other data types, we remove the label file entirely, as the pixel array cannot be loaded reliably via pydicom.

## Results

**Fig. 5:**
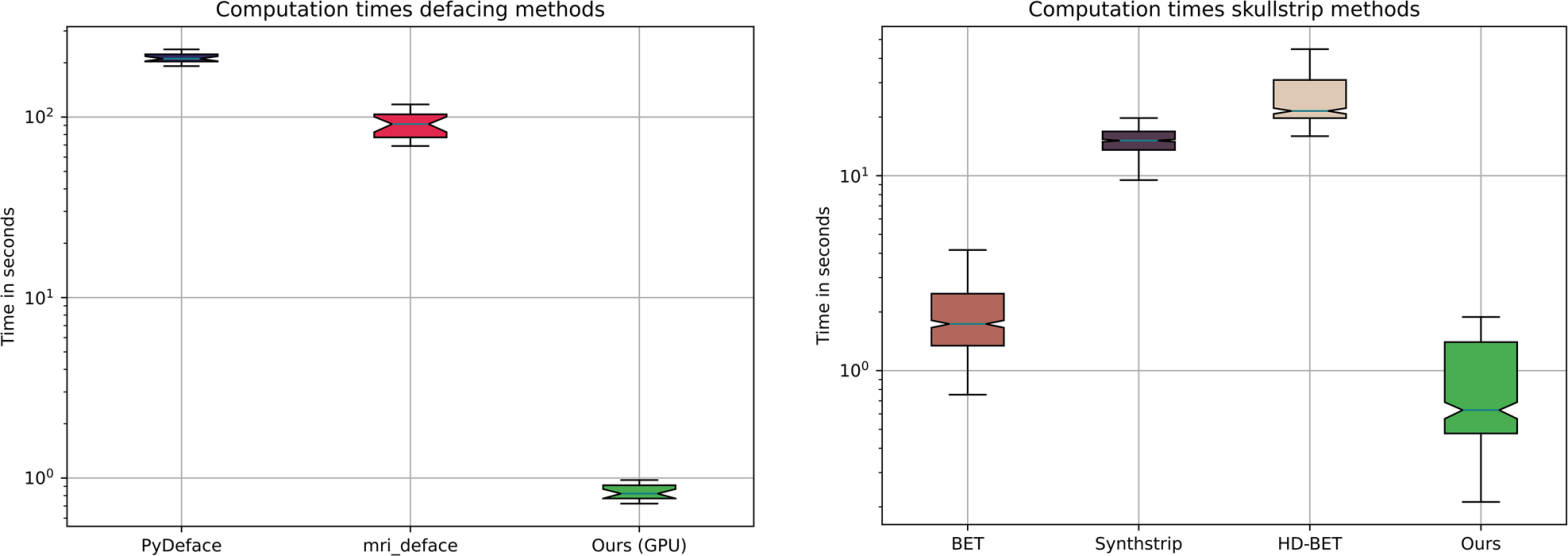
Computation time for defacing and skull-stripping of the compared methods. The proposed method is faster than the compared state-of-the-art algorithms. The *y*-axis is scaled logarithmically for better visibility

**Fig. 6:**
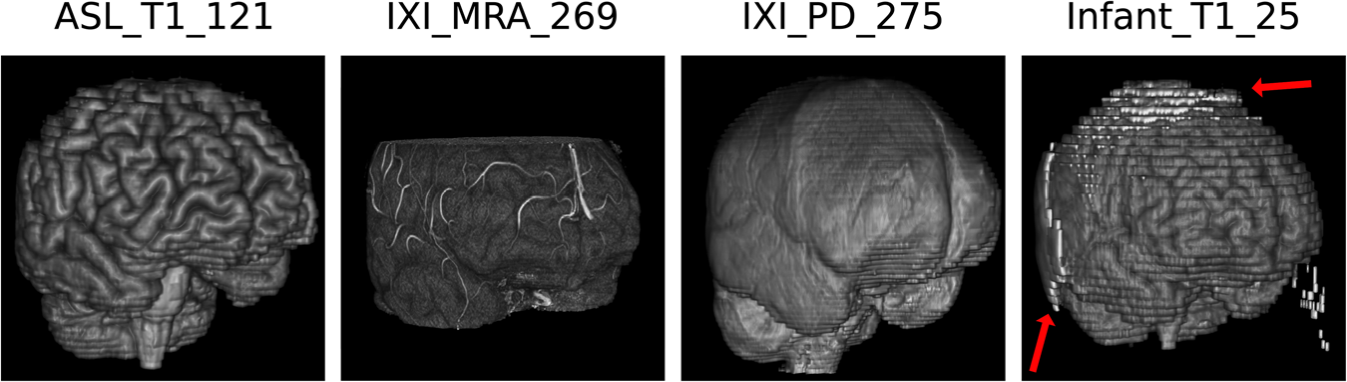
Skull-stripping results of the proposed algorithm. Shown are examples from the *Synthstrip* test dataset, including T1, magnetic resonance angiography (MRA), proton density (PD) and infant T1 scans. The proposed methods produce sound results, but might struggle with parts of infant brains

Both proposed algorithms for defacing and skull-stripping perform similarly to the compared state-of-the-art algorithms, while significantly reducing the computation time. The proposed defacing algorithm can reduce the computation time per volume by up to 260 times, to an average time of 0.88 ± 0.15 s, compared to 233.57 ± 59.87 s needed by *pydeface (*Table 2*)*. Even though *mri-deface* can reduce the computation time per volume to 95.59 ± 18.00 s, it still takes on average 105 times longer than the proposed method, while achieving a worse defacing score of 74.38 ± 17.24% in comparison to 80.62 ± 13.95%. The calculation time of the proposed method benefits from GPU support, but also reaches better times on CPU-only devices. A full overview of the results can be seen in Table 2. While *mri-deface* struggles with head scans of infants, *pydeface* and the proposed algorithm achieve sound results also on these types of scans. The *pydeface* algorithm removes the shoulder region of most of the scans, while the proposed method does not remove any additional body regions to the face. Table 3 shows the results of different skull-stripping algorithms in comparison to the proposed tool. While the proposed method outperforms the commonly used algorithm BET, it reaches similar results to *Synthstrip* and *HD-BET*. Nevertheless, the proposed method again outperforms both methods in terms of computation times, being 20 times faster than *Synthstrip*, with 0.77 ± 0.19 s against 15.34 ± 2.62 s per volume (Table 3). Figure 5 shows the computation time advantage of the proposed tool. For better visibility, we used a logarithmic scale. Exemplary results of the proposed skull-strip algorithm are shown in Fig. 6. The test dataset covers a wide range of modalities, including T1, magnetic resonance angiography (MRA), proton density (PD) and infant brain scans. The proposed method is able to perform the task of skull-stripping on all of these different modalities, but struggles with infant brain scans, leaving some skull residues in the output. The proposed text removal method reaches sound results, with a de-identification score of 83.59% on the tested ultrasound images. The scan is only counted as de-identified if all the text present in the image is removed. An example is shown in Fig. 4.

**Table 2:** Defacing scores and computation times per volume for different defacing algorithms

Method	Defacing score (%) ↑	Computation time (s) ↓
mrideface (CPU)	74.38 ± 17.24	95.59 ± 18.00 (× 108.6)
pydeface (CPU)	76.43 ± 14.53	233.57 ± 59.87 (× 265.4)
Ours (CPU)	**80.62** ± **13.95**	**25.07** ± **3.14 (× 28.5)**
Ours (GPU)	**80.62** ± **13.95**	**0.88** ± **0.15**

The computation time is given per average volume. While mri-deface and pydeface can only be used on CPUs, the proposed method can be used on GPU devices

The bold scores indicate an improvement over the current state of the art

**Table 3:** DICE score and computation times per volume for different skull-stripping algorithms

Method	DSC (%) ↑	Computation time (s) ↓
BET (CPU)	84.95 ± 14.22	4.15 ± 0.79 (× 5.4)
Synthstrip (GPU)	96.85 ± 1.03	15.34 ± 2.62 (× 19.9)
HD-BET (GPU)	95.10 ± 2.87	24.26 ± 5.90 (× 31.5)
Ours (GPU)	94.00 ± 3.61	**0.77** ± **0.19**

The computation time is given per average volume. While BET can only be used on CPU, Synthstrip, HD-BET and the proposed method offer GPU support

The bold scores indicate an improvement over the current state of the art

Moreover, the metadata de-identification was verified by a human tester on a dataset comprised of 410 files (DICOM and DICOM-WSI files). All specified metadata was correctly modified according to the specified DICOM profile.

## Discussion

We present a de-identification tool, which takes on the challenge of combining multiple de-identification steps in one tool, making it easy for clinicians and researchers to de-identify a wide variety of medical imaging data, without the need for different tools. By focusing on better computation times, we are able to speed up the process of de-identification, enabling real-time data processing, while achieving state-of-the-art (SOA) results. Nevertheless, our tool comes with limitations. One being the limited amount of training data and preprocessing steps. While the current SOA algorithms often perform an extensive preprocessing before defacing or skull-stripping, we almost completely do without these steps, with the goal of reducing the computation time. While we are able to reduce the computation times by up to 20 times in skull-stripping with GPU support, we sacrifice minor accuracy gains for the sake of computation time. This speedup is particularly important in clinical practice, when large amounts of data have to be processed on a daily basis. Additionally, the currently used text detection algorithm removes all text present in the data and does not differentiate between text information. This can also lead to the removal of useful information for downstream tasks. In research and clinical practice, the sole usage of JP(E)G or PNG files is uncommon and additional DICOM files, including the necessary metadata, are often used alongside. While the use of *Tesseract* is common practice, there are more advanced text detection algorithms that could be implemented in a future version of the proposed tool, such as the *Document understanding transformer* (Donut) by Kim et al [24]. Current state-of-the-art text detection approaches have become very sophisticated and reliable; however, they should not be employed without supervision or testing on data from the respective domain. Edge cases where current approaches fail can be encountered. Likewise, the de-identification of DICOM files can fail whenever manually editable string tags are preserved, as they can contain patient information that was entered mistakenly. If necessary, this can be prevented by choosing a DICOM profile that removes all string tags. The proposed WSI de-identification tool currently only supports DICOM-WSI files. This will be extended to further data types in the future.

## Conclusion

In this work, we propose a novel de-identification tool that combines metadata anonymization, defacing, skull-stripping and text removal in one framework. We give researchers and clinicians a tool that focuses on computation time and ease of use to enable them to perform real-time de-identification on a wide range of medical imaging data. While achieving computation time speedups of up to 260 times in comparison to commonly used tools, we achieve state-of-the-art results with a simple CLI tool that can be integrated into existing data processing pipelines. The proposed tool makes the tedious task of installing multiple different de-identification tools superfluous.

## Abbreviations

BET: Brain extraction tool
CLI: Command line interface
DICOM: Digital imaging and communications in medicine
NIfTI: Neuroimaging Informatics Technology Initiative
WSI: Whole slide image
